# Diseases of Children

**Published:** 1906-04-21

**Authors:** 


### DISEASES OF CHILDREN.

THERE are certain affections of infancy and childhood of which vomiting is the chief symptom. A good deal of mystery still hangs around the affection known as "**periodic or cyclical vomiting.**" It is characterised by the sudden onset of vomiting or regurgitation of food in a child apparently in good health. There is no nausea, the vomited matter is usually watery, but later may become bilious, and the prostration is not usually great. Such an attack may last from two to five days and may be followed by an interval of good health until the next attack, some days or weeks or months later. The whole subject has recently been discussed by Dr. Vincent Dickenson,[1] who does not find any of the current theories as to the pathology very convincing. Comby regards the condition as a manifestation of the arthritic diathesis. Snow and Whitby believe that it is a gastric neurosis, and think that hyperacidity and the absorption of ptomaines can precipitate the attack. A grave acid intoxication is the basis according to Edsall. This view is supported by the fact that diacetic acid is present in the urine, and that benefit is said to follow the use of alkalies in full doses. Griffith considers the affection to be of toxic origin, the poison being formed in the intestines or other tissues. Mery sees in constipation the root of all the evil. Richardière attributes the attacks to insufficient function of the liver, having been struck by the frequency with which hepatic symptoms predominate. Hutinel supports this hepatic theory, and adds that under normal conditions toxins formed in the system are altered by the liver and rendered harmless, but in the presence of renal insufficiency this toxin destruction does not take place and vomiting is induced. Marfan regards it as a distinctly family affection. Broca sees in these attacks a manifestation of an undiscovered chronic appendicitis, a theory which is disproved by the occurrence of attacks in patients after removal of the appendix. Summing up the subject, Dr. Dickenson says that the disease seems to be the effect of some toxic substance, the product of a defective metabolism, which accumulates in the tissues, and which, acting on an unstable nervous system whose limit of tolerance is reached, suddenly produces an attack. Apart from the alkaline treatment referred to above, little benefit has resulted from the various therapeutical measures tried. Another affection in which vomiting is the chief symptom is **congenital pyloric stenosis.** The vomiting usually comes on during the first few weeks of life and persists in spite of every care in dieting. At a later stage the cardinal signs are visible peristalsis of the stomach and a palpable enlargement of the pylorus. As regards the medical treatment of these cases, Dr. Newman Neild [2] records two cases which he has treated successfully with opium. The infants were aged five weeks and eight weeks respectively, and in both cases the pylorus could be felt, peristalsis of the stomach was present, and the other signs of the affection were clear. One-eightieth of a minim of laudanum was ordered to be given twenty minutes before each feed. In one case the dose was increased to one-fortieth of a minim. The result in both cases was a rapid diminution in the vomiting and a gain in weight, followed by complete recovery. No alteration was made in the diet, and the cases were both treated as hospital out-patients. Dr. Crozier Griffith [3] records a case which recovered without operation. Vomiting occurred on the sixteenth day of life and continued. Eight days later the stomach was evidently dilated and active peristaltic movements could be seen, the waves moving slowly from left to right. There was an absence of fæcal matter in the stools, and emaciation was increasing, notwithstanding careful dieting. Lavage was practised daily. Gradual improvement took place. The author suggests that in this case the cause of the obstruction was chiefly spasm, the action of which was aided by swelling of the mucous membrane, dependent on the irritated state of the whole gastro-intestinal tract. He believes that there was stenosis, apparently complete, for ten days, and more or less obstruction for weeks afterwards.

The surgical treatment of infantile hypertrophic stenosis of the pylorus has been recently reviewed by Mr. H. J. Paterson.[4] The operations carried out have been pylorectomy, gastro-jejunostomy, pyloroplasty, and pylorodiosis or Loretta's operation. Pylorectomy is to be dismissed as unnecessarily severe and unjustifiable. Out of 25 cases of gastro-jejunostomy, 14 died and 11 recovered. As regards three of the successful cases, the condition of whom is known some years after the operation, the children are described as being well in every way. The inference is that the operation has no effect on the metabolic processes of the body. Pylorodiosis has been performed 21 times with 6 deaths and 15 recoveries. Relapse has occurred in several of the cases. Nine cases of pyloroplasty have been recorded, of whom 6 recovered and 3 have died. In several of the cases there has been trouble with the feeding after the operation. Many operators have found that the operation is by no means an easy one. The writer is inclined to agree with Mr. Stiles in preferring the operation of gastro-jejunostomy for the following reasons: (1) It is better to operate on normal than on morbid tissues. (2) Feeding can be commenced at once after gastro-jejunostomy. (3) If the anterior operation be performed it can be completed within twenty-five minutes. (4) The remote results are highly satisfactory.

"**Food fever**" is the name given by Dr. Eustace Smith [5] to a disorder which comes on suddenly, is accompanied by signs, more or less pronounced, of digestive disturbance, lasts in its acute form for several days, and may linger on in a modified degree for several weeks. The subjects of this affection are neurotic children between the ages of 3 and 12 years. During an attack the temperature varies from 101° to 105°. Under treatment by a mercurial purge the temperature falls, but soon rises again. The patients make no complaint of bodily discomfort, but are fretful and irritable in the day, and at night lie awake or sleep badly, grunting and groaning and tossing from side to side. Appetite is usually lost. These attacks are often repeated every five or six weeks, and nutrition begins to suffer. The frequent recurrence of fever is the chief cause of anxiety, especially as the attacks come on quite suddenly and without obvious cause. Vomiting may be a marked feature, and the author is of opinion that many of the cases of "periodic" or "cyclical" vomiting are of this nature. The explanation of the attacks is given as an auto-intoxication due to certain articles of food. The treatment consists in removing from the dietary all excess of carbohydrates and all materials capable of unwholesome fermentation in the bowels. Starches and sweets are to be given with caution, and the ordinary milk puddings are to be strictly forbidden. Milk is harmful, and acid fruits such as baked apples, grapes, oranges, and lemonade must be condemned. The proper diet consists of mutton, poultry, white fish, well-boiled green vegetables, and eggs. Plenty of butter with stale bread, toast, and rusks are allowed. Attention also must be paid to the avoidance of all chilling of the surface of the body, either indoors or out of doors, which is often the precursor of an attack.

An interesting innovation was carried out last summer at the Great Ormond Street Hospital, when a special ward was set aside for infants suffering from **diarrhœa**. In describing the results obtained, Dr. F. E. Batten [6] remarks that, realising the risk of infection spreading, the authorities arranged for the nursing being carried out on the same lines as for typhoid fever. Two or more nurses attended to the removal of the soiled diapers and did not feed the children. In all, 120 infants suffering from diarrhœa were admitted, and of these 30 may be said to have presented the features of acute infective diarrhœa. Of these 30 cases 12 recovered and 18 died, a mortality which is probably quite up to the average. In some of these death occurred during the acute stage, and in others death followed from some toxic sequela affecting the digestive, the urinary, the nervous, or the vaso-motor system. Success in the cases which recovered is traced to the fact that they were of less severity and came under treatment early in the course of the disease. The important elements in the treatment were (1) hypodermic injections. Strychnine injections (half a minim of the liquor strychninæ) were given every four hours, while injections of brandy or ether were discouraged. (2) Subcutaneous saline injections, consisting of four ounces of normal saline fluid, at a temperature of 150° F., were slowly injected into the subcutaneous tissues. (3) Hot baths at a temperature of from 100° F. to 110° F. were found very useful. (4) Stomach wash-out. (5) Feeding: All milk was stopped, and albumen-water—the white of one egg to four ounces of water—was administered every two hours in amounts varying from one to two ounces according to age. The next stage in the dietary was whey and albumen water, then citrated milk, and then peptonised milk. The employment of raw meat juice, meat extracts, and various proprietary preparations was not found to be of any benefit. (6) Drugs: During the acute stage calomel and castor oil were alone of much value. In the later stages acids, alkalies, bismuth, and opium have their uses, and bromides in cases of restlessness.

[1] Brit. Jour. Child. Dis., March, 1906. [2] The Lancet, Nov. 25, 1905, p. 1543. [3] Arch. of Pediat., Oct., 1905, p. 721. [4] The Lancet, March 3, 1906, p. 577. [5] Brit. Med. Jour., Feb. 10, 1906. [6] Clin. Jour., Jan. 3, 1906, p. 177.

*(To be continued.)*

